# An LCA of the Pelamis wave energy converter

**DOI:** 10.1007/s11367-018-1504-2

**Published:** 2018-07-23

**Authors:** R. Camilla Thomson, John P. Chick, Gareth P. Harrison

**Affiliations:** School of Engineering, Institute for Energy Systems, University of Edinburgh, Edinburgh, EH9 3DW UK

**Keywords:** Carbon footprint, Embodied energy, Environmental impacts, Life cycle, Renewable energy, Wave energy

## Abstract

**Purpose:**

To date, very few studies have attempted to quantify the environmental impacts of a wave energy converter, and almost all of these focus solely on the potential climate change impacts and embodied energy. This paper presents a full life cycle assessment (LCA) of the first-generation Pelamis wave energy converter, aiming to contribute to the body of published studies and examine any potential trade-offs or co-benefits across a broad range of environmental impacts.

**Methods:**

The process-based attributional LCA was carried out on the full cradle-to-grave life cycle of the Pelamis P1 wave energy converter, including the device, its moorings and sub-sea connecting cable up to the point of connection with the grid. The case study was for a typical wave farm located off the north-west coast of Scotland. Foreground data was mostly sourced from the manufacturer. Background inventory data was mostly sourced from the ecoinvent database (v3.3), and the ReCiPe and CED impact assessment methods were applied.

**Results and discussion:**

The Pelamis was found to have significantly lower environmental impacts than conventional fossil generation in 6 impact categories, but performed worse than most other types of generation in 8 of the remaining 13 categories studied. The greatest impacts were from steel manufacture and sea vessel operations. The device performs quite well in the two most frequently assessed impacts for renewable energy converters: climate change and cumulative energy demand. The carbon payback period is estimated to be around 24 months (depending on the emissions intensity of the displaced generation mix), and the energy return on investment is 7.5. The contrast between this and the poor performance in other impact categories demonstrates the limitations of focussing only on carbon and energy.

**Conclusions:**

The Pelamis was found to generally have relatively high environmental impacts across many impact categories when compared to other types of power generation; however, these are mostly attributable to the current reliance on fossil fuels in the global economy and the early development stage of the technology. Opportunities to reduce this also lie in reducing requirements for steel in the device structure, and decreasing the requirements for sea vessel operations during installation, maintenance and decommissioning.

**Electronic supplementary material:**

The online version of this article (10.1007/s11367-018-1504-2) contains supplementary material, which is available to authorized users.

## Introduction

The drive to decarbonise electricity supplies around the world, in an ongoing effort to mitigate climate change, has encouraged an increase in renewable energy generation. In the UK, it is expected that a virtually complete decarbonisation of the electricity sector will be required by 2050 to meet national emissions reduction targets (Wiedmann et al. 2011). Marine energy (wave and tidal) is likely to contribute significantly to this, with an estimated potential installed capacity of 30 to 50 GW (BEIS 2013).

While wave energy sources are inherently low-impact, energy is consumed and pollutants are emitted during the construction, operation and decommissioning of the energy converters. This has led to questions over whether these new technologies will deliver a net reduction in greenhouse gas (GHG) emissions and a viable energy return on energy investment (EROI). In order to answer this, it is necessary to identify the life cycle GHG emissions and energy consumption of the converters, resulting in a handful of studies based on life cycle assessment (LCA) methodology (Douglas et al. 2008; Parker et al. 2007; Soerensen and Naef 2008; Uihlein 2016). The small number of existing studies, however, coupled with the significant variation in design of marine energy converters, makes it difficult to corroborate results or draw definitive conclusions about the impacts of wave energy. Furthermore, all but one of the existing studies focus solely on GHG emissions and embodied energy and may overlook potential trade-offs or co-benefits between environmental impacts (WRI and WBCSD 2011).

The analysis presented here is a full life cycle assessment of the first-generation Pelamis wave energy converter (WEC). The work aims to contribute to the body of published studies of marine energy converters and examine any potential trade-offs or co-benefits by setting the GHG emissions and embodied energy in the context of a broader range of environmental impacts.

## Background

### Wave energy converters

A number of different wave energy converters are under development, with the European Commission’s Joint Research Centre’s (JRC) ocean energy database currently containing details of over 100 different designs (Uihlein 2016). These designs all vary widely, and are usually classified into a number of different broad categories: the IPCC suggests three—oscillating body systems, oscillating water columns and overtopping devices—while the JRC suggests eight—dividing oscillating body systems into attenuator, point absorber, oscillating wave surge, pressure differential and rotating mass systems and also considering “other” devices, to better reflect more recent developments in this sector (IPCC 2011; Uihlein 2016). Furthermore, these devices can be shore-mounted, floating or seabed mounted, adding further variation to the designs.

### The Pelamis WEC

The Pelamis WEC is an example of a floating oscillating body system of the attenuator type, as it extracts energy from the oscillation induced by the wave motion on separate sections of tube. It is a semi-submerged ‘snake-like’ offshore device, developed by Pelamis Wave Power Ltd. (PWP), and initially emerged as one of the most promising devices in the marine energy sector: it was the first WEC to be installed at a commercial scale, with the P1 model successfully installed at Aguçadoura, Portugal, in 2008 (Aquaret 2008).

**Fig. 1:**
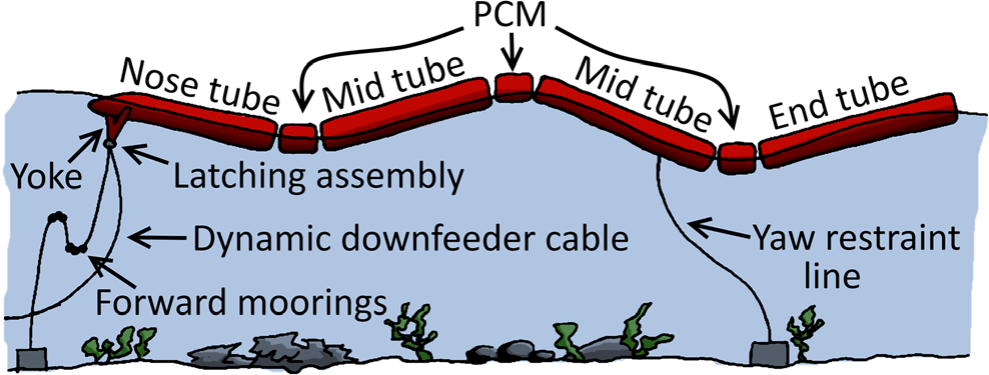
Sketch of Pelamis components

Figure 1 shows the principal components of the Pelamis. It is 120 m long, 3.5 m in diameter and rated at 750 kW. It has four cylindrical steel tube sections linked by three power conversion modules (PCMs) at the hinged joints. The moorings allow the Pelamis to face into the oncoming waves, and the joints flex vertically and horizontally as the wave front passes. This motion is resisted by hydraulic rams, which pump high-pressure oil into accumulators that are drained through hydraulic motors to drive induction generators. The hydraulic power take-off, generators and control equipment are all housed within the PCMs, while the nose tube, tapered at one end to allow the device to cut through large waves, houses the switchgear and transformer to collect the power for export to shore. The resistance of the rams can be tuned to provide a resonant response in small sea states to maximise power capture, and can also assist in protecting the device from potentially damaging storm waves. A Y-shaped element (yoke) connects the nose tube to the mooring and cabling system; this has a quick-release tethering system to allow for rapid attachment and detachment. Sand ballast in the main tube sections optimises the buoyancy. Further information can be found in Henderson (2006).

### Environmental impacts of wave energy

Very few studies have been carried out that examine the life cycle environmental impacts of WECs, with only seven having been identified by the authors. Of these, three (Banerjee et al. 2006; Banerjee et al. 2005; Carbon Trust, 2006) are first-order estimates of the carbon footprint and embodied energy based only on the mass of steel (Banerjee, et al. (2006) includes copper) and do not capture the full impacts. Another three are partial life cycle inventories of wave energy converters: a CO_2_ and embodied energy audit of the Pelamis (Parker et al. 2007), a study with a similar scope on a seabed-mounted oscillating wave surge converter called the Oyster (Walker and Howell 2011), and a study on the Wave Dragon floating overtopping device that also considers emissions of methane (Soerensen and Naef 2008). Only one full LCA of WECs has been identified, although this is very comprehensive as it includes all of the WECs stored in the JRC ocean energy database (Uihlein 2016) (note that Uihlein also considers a preliminary version of this study in his discussion, but the analysis presented here has been significantly extended and updated (Thomson et al. 2011).)

These studies have not necessarily agreed on conclusions. Parker et al. (2007) and Walker and Howell (2011) found that the carbon footprint and embodied energy for the Pelamis and Oyster were similar (23 g CO_2_/kWh and 293 kJ/kWh for the Pelamis and 25 g CO_2_/kWh and 236 kJ/kWh for the Oyster) but Uihlein (2016) estimated the potential climate change impacts for these types of device to be higher, at 44 and 64 g CO_2_ eq/kWh for an attenuator or oscillating wave surge device, respectively. This discrepancy may well be due to the specific scenarios considered, and the Pelamis and Oyster devices do have some similarities as large, steel structures with hydraulic power take-off systems. In contrast, Soerensen and Naef (2008) found the climate change impacts of the Wave Dragon to be only 13 g CO_2_/kWh, but this device is predominantly concrete. The significant variation between designs of marine energy converters makes it challenging to draw conclusions on the environmental impacts of the sector from the small number of studies that have already been carried out.

## Methods

### Goal and scope

The goal was to carry out a detailed LCA of the first-generation Pelamis P1 for a single case study installation scenario. The system boundary encompasses the full cradle-to-grave life cycle including the device, its moorings and sub-sea cable up to the connection with the grid. All downstream electrical components were excluded. Every stage of manufacture, operation and decommissioning was examined. The functional unit is 1 kWh of output electrical power, with a reference flow of one Pelamis.

This study considers a generic case of a single Pelamis P1 based on manufacturer’s data and a fixed scenario for manufacture, assembly and deployment of the device at a location off the north-west coast of Scotland. Manufacture of the steel tube sections and final assembly takes place at a steel fabrication yard on the nearest coast; the PCMs housing the complex power take-off equipment are assembled near Edinburgh, ~ 420 km away; once completed, the Pelamis is towed to the installation location, around 320 km from the fabrication yard, implying a travel time of 24 h at 7 knots. Later versions of the device and different installation scenarios will have different impacts.

The average annual energy production of a single device at the site is estimated to be 2.97 GWh/year over the 20-year design life, corresponding to a capacity factor of 45%; the installation at Aquaçadoura performed as expected, so this assumption is considered valid (Parker et al. 2007; PWP 2011). Unless otherwise specified, it is also assumed that all components are manufactured in the UK and subject to UK energy statistics and transport distances.

In line with other published LCA studies of renewable generators, the impacts of grid integration are excluded, other than to estimate the emissions displacement. While production patterns will differ from wind, it is expected that the system effects of wave power will be broadly similar on the British grid (Thomson 2014); this issue merits a separate, detailed study.

### Tools

The analysis was carried out using SimaPro (version 8.3 PhD), with life cycle inventory data sourced from the ecoinvent database (version 3.3). A comprehensive list of all processes and materials selected from ecoinvent is provided in Section S1 of the Electronic Supplementary Material.

The life cycle impact assessment (LCIA) was carried out with the ReCiPe Midpoint method, hierarchist version, with European normalisation (ReCiPe, 2016) along with the Cumulative Energy Demand (CED) method (Goedkoop et al. 2008; Hischier et al. 2010).

### Input data

All foreground data for the materials and processes in the life cycle were from data derived from PWP’s own records for the study by Parker et al. (2007). All background data was ultimately from the ecoinvent database, with some assumptions required for a few manufacturing and shipping processes, as described below. A detailed summary of the key parameters and all inputs to this analysis is given in the Electronic Supplementary Material, Section S1, including a flowchart (Fig. S1.2).

#### Materials and manufacture

A mass-based analysis was carried out for the structure, hydraulic system and mooring components, with a breakdown of the materials used in Table 1; inventory data was all sourced from ecoinvent v3.3. Pelamis is largely constructed from steel, which is cut and welded to shape before being sand blasted and painted with a corrosion-resistant paint. As detailed in the Electronic Supplementary Material, Section S1, inventory data for material processing was sourced from ecoinvent, but where this was not available the following approximations were made:

- Oxy-acetylene flame cutting was approximated using data for gas welding. The data from PWP is quantified by the area of material removed with welding quantified by length; assuming a typical weld is 20 mm wide, 1 m^2^ of material removed is equivalent to a 50-m weld. The weld material in ecoinvent is minimal and was disregarded.
- Sand blasting was approximated from published data on typical air supply pressure for abrasive blasting (Kalpakjian et al. 2008), manufacturer’s data for air volume requirements (Axxiom 2008), quantity of abrasive (Jiven et al. 2004) and ecoinvent data on particulate emissions (Classen et al. 2009). The resulting process and uncertainty estimate is detailed in the Electronic Supplementary Material, Table S1.5.
- Data sheets on the ‘glass-flake’ paint used and ecoinvent data allowed an inventory to be created (Hempel [Bibr CR22][Bibr CR23]; Hempel 2010b; Hempel 2010a; Hempel 2010b); this is detailed in the Electronic Supplementary Material, Tables S1.5 and S1.6.
- Paint is applied with an airless spray and Parker et al. (2007) estimated an overall 1 mm thickness; the process was approximated using coverage information from the manufacturer (Hempel, 2007), and compressed air requirements from manufacturer’s data (Graco 2010).

**Table 1:** Material quantities in the Pelamis P1, not including pre-fabricated components such as fixings and electronics

Stock material	Mass (kg)
Steel	561,954
Sand	475,722
Stainless steel	550
Nylon 6	416
Polyurethane	343
Glass reinforced plastic (GRP)	90
PVC pipe	55

The Pelamis also contains many pre-fabricated components evident in the bill of materials containing quantities and costs (this cannot be reproduced here due to confidentiality). Of the pre-fabricated components in the study by Parker et al. (2007) only items contributing at least 10% to the total cost, embodied carbon or energy were included: the transformer, main generators and switchboard. Excluded components are estimated to contribute less than 1% to the total impacts. Data for typical high-voltage transformers and switchgear is not included in ecoinvent, so simplified materials-only models were created from manufacturer’s data: see Electronic Supplementary Material, Section S1.3 (ABB, 2001, ABB, 2007, ABB, 2010).

#### Assembly and installation

The case study scenario described in Section 3.1 involves most manufacturing and assembly taking place at a fabrication yard. As ecoinvent market data already includes average data for transportation of materials, no additional transportation is included for items manufactured at the fabrication yard. Transport impacts were considered, however, for the complex components of the PCMs housing the hydraulics. Based on PWP data, typical ecoinvent mass-distance data was used for freight transport (Table 2, and the Electronic Supplementary Material, Section S1.2). Where data was not provided, estimates were made (indicated in *italics*) and components with no specific source were assumed to come from the centre of the UK by population, ~ 540 km away by road (Dorling and Atkins 1995).

**Table 2:** Transport data for PCM components. Estimates are shown in italics

Component	Total mass	Source	Distance	Transport
	(kg)	Location	(km)	Method
Panels	*20*	Scotland	*100*	Road haulage
MG Set	60	Scotland	106	Road haulage
Structural shell	23,207	Scotland	130	Road haulage
Hydraulic rams	5800	England	510	Road haulage
Reservoirs and oil	*2620*	UK	*540*	Road haulage
Manifold and hoses	450	UK	*540*	Road haulage
Misc items	*160*	UK	*540*	Road haulage
Heat exchanger	*100*	Holland	*750*	Cargo ship
Accumulator pack	3000	Wales	722	Road haulage
Bellow	*100*	China	*18,000*	Cargo ship

PWP provided data on the PCM assembly in the form of hours of operation of fork-lifts and overhead cranes. Impacts were approximated using only the fuel and energy consumption using ecoinvent data, as detailed in the Electronic Supplementary Material, Table S1.9.

The completed PCMs are transported to the fabrication yard for final assembly and installation. A range of specialist sea vessels are then required to install moorings and power cabling, carry out sea trials, tow and install the device. Detailed sea vessel requirements and fuel consumption data were provided for a farm of 30 devices, with requirements per Pelamis summarised in Table 3. Resource use and emissions were approximated by scaling ecoinvent data for a freight ship to match the fuel consumption detailed in the Electronic Supplementary Material, Table S1.9.

**Table 3:** Total sea vessel operations for 1 Pelamis. Activities include installing and recovery of moorings and power cabling, sea trials, towing to site, latching, unlatching and inspections

Sea vessel	Fuel consumption (l/day)	Total days of operation
Installation		
Barge	290	11.8
Multicat	1710	23.8
Tug	1490	11.8
Annual maintenance		
Tug	1490	4
Inspection vessel	500	1.3
Decommissioning		
Barge	290	2.5
Multicat	1710	8.5
Tug	1490	2.5

#### Operations and maintenance

During operation, the Pelamis is remotely monitored and controlled by an onshore computer, likely to have minimal environmental impacts; therefore, none were considered for the operational stage. Annual maintenance requirements were estimated by PWP and are understood to be conservative, with the key aim of confirming and ensuring survivability. Most maintenance activities are expected to take place in port, but PWP was only able to provide data for the sea vessel operations associated with this (Table 3). This includes two unlatching/re-latching operations per year, including detachment from mooring, tow to shore and redeployment, and six inspections of the moorings using remotely operated vehicles. These sea vessel operations are again approximated by scaling ecoinvent data for a freight ship (Electronic Supplementary Material, Table S1.9). Due to uncertainty over the maintenance requirements, and following the same assumptions as Parker et al. (2007), no allowance was made for processes or materials required for replacement of parts; this may underestimate impacts from this stage but is expected to be modest.

#### Decommissioning and disposal

It is expected that decommissioning will require sea vessel operation to unlatch the Pelamis, tow it back to shore and recover all mooring hardware. The impacts of this were again estimated by scaling ecoinvent data for a freight ship, based on PWP fuel consumption data (Table 3). Due to uncertainty over the processes involved, no allowances were made for the impacts of dismantling the device.

Waste is expected to be divided into two streams with most steel being recycled and the remainder going to landfill. The recycling rate is taken as 90% to allow for incomplete recovery of the steel. No end-of-life recycling is considered for other metals and recyclable materials. The selected ecoinvent data for landfill waste treatment is detailed in Table S1.10 of the Electronic Supplementary Material, and recycling impacts are discussed in Section 3.4.

### Recycling allocation

The Pelamis WEC is manufactured from a significant quantity of steel, and it is likely that much of this will be recovered and recycled at the end-of-life. Existing LCA studies of renewable energy converters often include a credit for recycling materials at the end-of-life, but the allocation method is not always clear (Ardente et al. 2008; Douglas et al. 2008; Kannan et al. 2005; Martínez et al. 2015; Parker et al. 2007; Walker and Howell 2011), and it is possible that recycling benefits could be routinely double-counted.

Version 3.3 of the ecoinvent database applies three different models for allocating co-products within the background data: allocation at the point of substitution (APOS), recycled content and consequential (Wernet et al. 2016). APOS sees flows allocated relative to their ‘true value’, which is the economic revenue corrected for some market imperfections and fluctuations (Weidema et al. 2013). This is considered by some to enable most consistent allocation in attributional LCA, such as that presented here (Schrijvers et al. 2016).

In order to correctly allocate the inventory flows for steel recycling at the end of life, however, it is preferable to use the same allocation method as the background data. Although the APOS method employed in the ecoinvent database has been detailed in Wernet et al. (2016), it is not described mathematically, and it is therefore unclear how the impacts of recycling have been divided in the partial life cycle (cradle-to-gate or gate-to-grave) data within ecoinvent. This makes it challenging to extend into the foreground section of the life cycle without the risk of double-counting.

This analysis, therefore, applies the recycled content approach for both foreground and background processes. This is a simple cut-off allocation method where the end-of-life recycling processes are considered to be outside the system boundary, and no recycling credit is considered.

### Sensitivity and uncertainty analysis

In order to examine any uncertainty introduced by data quality, a Monte Carlo analysis was carried out. The ecoinvent database contains uncertainty ranges for all background data, generally modelled as a lognormal distribution where the square of the geometric standard deviation covers the 95% confidence interval. This standard deviation is estimated from an uncertainty factor with additional factors from data quality indicators via a pedigree matrix (Weidema et al. 2013). For consistency, the uncertainty of all foreground data and data sourced from elsewhere was estimated using the same process. The only exception was for some materials in the paint, where the datasheets provided uncertainty ranges. The selected uncertainty indicator scores for all input data are included in the Electronic Supplementary Material, Section S1.

A simple sensitivity study was also carried out to see how impacts varied when individual parameters were varied from their baseline. These were restricted to energy production and travel distances which are driven by location, and operating lifetime. These were varied by the ranges in Table 4 and enable others to extrapolate estimates of impacts to other locations. Far more sophisticated global sensitivity methods are available, e.g. Plischke et al. (2013).

**Table 4:** Ranges to test sensitivity of results to design life and factors relating to the installation location

Parameter	Original value	Range tested
Annual energy output, represented by capacity factor	45%	25–55%
Distance of installation location from fabrication yard	320 km	20–320 km
Distance to steel fabrication yard from Pelamis factory	420 km	210–630 km
Design life	20 years	10–30 years

## Results

### Life cycle impact assessment

The environmental impacts from both the ReCiPe and CED impact assessment methods are summarised in Table 5, along with the acronyms used for each impact category. The CED results are calculated for six types of primary energy carrier and as different concepts exist for characterising these, the resulting values may not be comparable across types. It has been assumed that they can be combined into a single value, while the complete results are given in Table S2.1 in the Electronic Supplementary Material.

**Table 5:** Results of LCIA and cumulative energy demand calculation

Impact category		
Climate change (CC)	35	g CO_2_ eq/kWh
Ozone depletion (OD)	3.7	μg CFC-11 eq/kWh
Photochemical oxidant formation (POF)	325	mg NMVOC/kWh
Terrestrial acidification (TA)	404	mg SO_2_ eq/kWh
Freshwater eutrophication (F Eut)	21	mg P eq/kWh
Marine eutrophication (M Eut)	14	mg N eq/kWh
Particulate matter formation (PMF)	184	mg PM10 eq/kWh
Human toxicity (HT)	33	g 1,4-DB eq/kWh
Terrestrial ecotoxicity (T Etox)	4.2	mg 1,4-DB eq/kWh
Freshwater ecotoxicity (F Etox)	906	mg 1,4-DB eq/kWh
Marine ecotoxicity (M Etox)	924	mg 1,4-DB eq/kWh
Ionising radiation (IR)	2.4	Bq ^235^U eq/kWh
Agricultural land occupation (ALO)	915	mm^2^a/kWh
Urban land occupation (ULO)	393	mm^2^a/kWh
Natural land transformation (NLT)	8.5	mm^2^/kWh
Water depletion (WD)	241	cm^3^/kWh
Metal depletion (MD)	26	g Fe eq/kWh
Fossil depletion (FD)	10	g oil eq/kWh
Cumulative energy demand (CED)	493	kJ/kWh

**Fig. 2:**
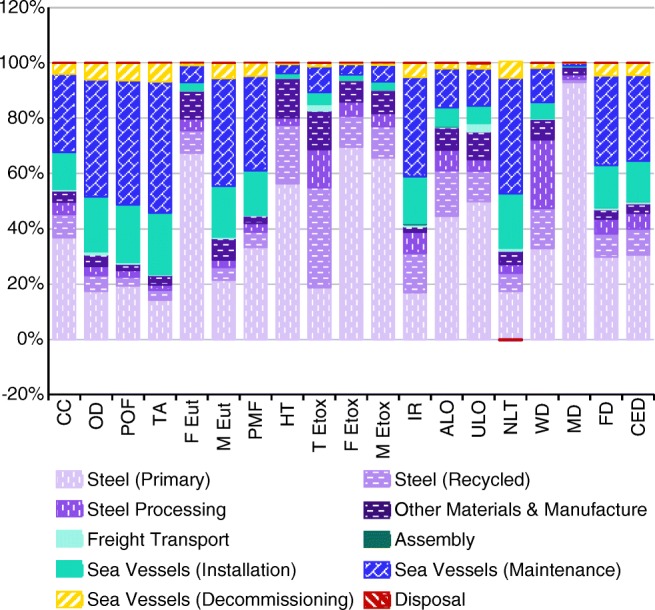
Breakdown of impacts by life cycle stage for each impact category. Acronyms are defined in Table 5

Figure 2 shows the contribution of different processes and life cycle stages to each impact category. Steel production and processing has a significant impact in virtually all categories, particularly freshwater eutrophication, human toxicity, freshwater and marine ecotoxicity and metal depletion. Most of the remaining impacts can be attributed to sea vessel operations, which are particularly significant in ozone depletion, photochemical oxidant formation, terrestrial acidification and natural land transformation. In contrast, the impacts of freight transport (conventional road haulage and container shipping), assembly and disposal processes are relatively small. It can also be seen that there is a very small negative impact at the waste disposal stage for natural land transformation. This is due to a credit for re-naturalisation of inert material landfill sites after closure, where the ecoinvent data has a share of the site being created from pasture and re-naturalised to forest; the ReCiPe method gives a greater natural land transformation impact/credit to forested land than pasture.

The relative contribution of the impacts from recycled steel when compared to primary steel is of interest. The recycled content of the global steel mix in the ecoinvent v3.3 data is 43% (ecoinvent 2016); however, this accounts for an average across all categories of only 23% of the total impacts of steel production. In several categories (climate change, photochemical oxidant formation, freshwater and marine eutrophication, particulate matter formation, freshwater and marine ecotoxicity, urban land occupation and metal depletion), the impacts of recycled steel are less than one third of the impacts of primary steel. There are, however, two categories where the impacts of recycled steel are higher than for primary steel: high terrestrial ecotoxicity impacts are introduced during the processing and transport of the scrap metal, while ionising radiation impacts are slightly higher due to a higher consumption of electricity from nuclear power stations.

Carbon and energy indicators are of particular interest for renewable energy converters. With a climate change impact of 35 gCO_2_ eq/kWh, the carbon payback period was estimated to be only 18 months for installation in 2006 or 24 months in 2017, assuming that the device offsets the average generation mix in the UK (473 gCO_2_ eq/kWh in 2006 (Ricardo-AEA, 2006) and 352 gCO_2_ eq/kWh in 2017 (BEIS and DEFRA 2017)); however, this is likely to be shorter due to it offsetting only the more carbon-intensive marginal generation mix, as discussed in Thomson et al. (2017). The EROI or energy ratio is found to be 7.3, with an energy payback period of 33 months.

### Sensitivity and uncertainty analysis

**Fig. 3:**
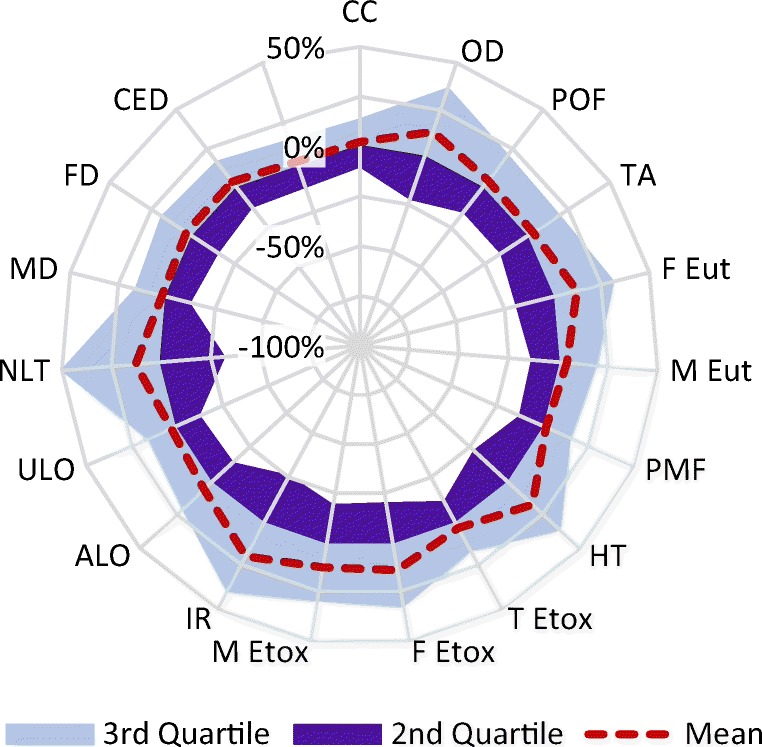
Interquartile ranges relative to the median for each impact category except water depletion. Acronyms are defined in Table 5. The range for water depletion is − 377/+ 315% of the median value

**Fig. 4:**
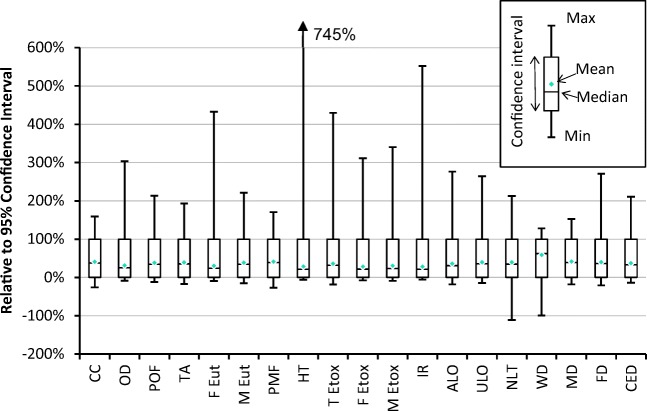
Full uncertainty ranges relative to 95% confidence interval for each impact category, with confidence interval set as 0–100%. Acronyms are defined in Table 5

The sensitivity of the results to uncertainty in the input data is illustrated in Fig. 3, with full numerical results given in Table S2.2 of the Electronic Supplementary Material. It can be seen that the interquartile ranges vary significantly across impact categories, with the lowest being for climate change, particulate matter formation, terrestrial ecotoxicity, urban land occupation, metal depletion and cumulative energy demand and the highest for natural land transformation and water depletion (the latter is too large to show on the chart). The highest impacts in all six of these categories are due to processes during steel manufacture, or for shipping fuel production and combustion, so further investigation is required to explain why the overall uncertainties are so much higher for two of them; however, these ranges are given relative to the median, and therefore the larger relative uncertainties may reflect a relatively low median value for that impact category. Typical 95% confidence intervals are around − 40%/+ 75%, and it can be seen in Fig. 4 that the overall ranges are typically less than five times this confidence interval, except for human toxicity and ionising radiation.

**Fig. 5:**
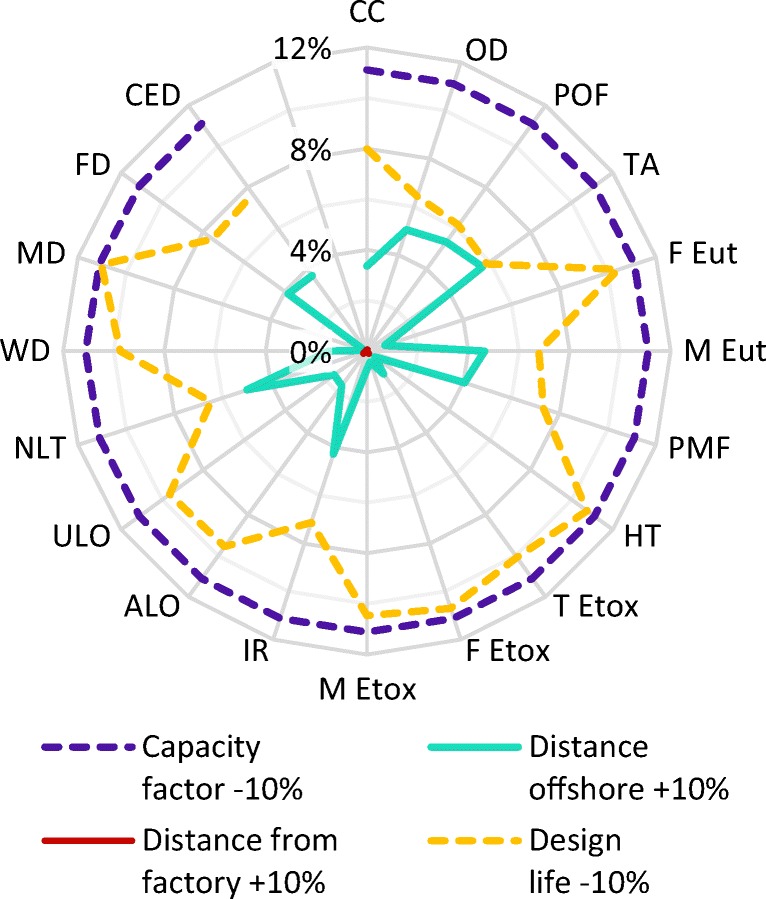
Sensitivity of results to ± 10% changes to key parameters for each impact category. Acronyms are defined in Table 5

**Fig. 6:**
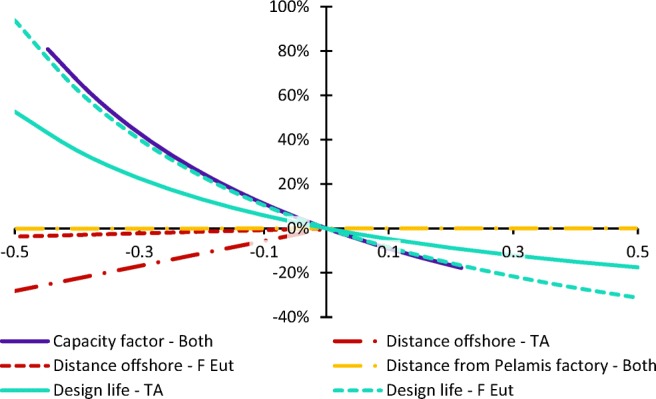
Sensitivity of terrestrial acidification (TA) and freshwater eutrophication (F Eut) to changes in key parameters

The sensitivity of the results to key parameters specific to the particular case study shown here is illustrated in Fig. 5, by showing the maximum positive change as a result in a change of ± 10%. The full results are given in Table S2.3 in the Electronic Supplementary Material, and the trends in sensitivity of two impact categories (terrestrial acidification and freshwater eutrophication) are illustrated in Fig. 6. Note that uncertainty distributions for all of these parameters were included in the uncertainty analysis; this sensitivity analysis is provided for additional information on the impact of the selected case study scenario on the results.

As might be expected, an increase in capacity factor or design life would reduce the overall environmental impacts, while a decrease in distances travelled would have the same effect. All impact categories are most sensitive to the estimated annual energy production (represented by capacity factor) and least sensitive to the distance from the Pelamis factory to the steel fabrication yard.

The sensitivity of the results to the estimated design life varies across impact categories, and is generally smaller than the sensitivity to annual energy production. The difference is greatest in impact categories most affected by the operation of sea vessels for maintenance purposes, such as terrestrial acidification (Fig. 6). This is due to the maintenance requirements being defined annually: the saving made by reducing the maintenance requirements offsets some of the increases in impacts caused by a reduction in design life, thus reducing the lifetime energy production and increasing the overall impacts per kilowatt hour. Terrestrial acidification impacts are partly due to the emissions of pollutants from combustion of shipping fuel, while freshwater eutrophication impacts mostly arise during steel production.

The results are also much more sensitive to varying the distance from the fabrication plant to the installation site than the distance from the Pelamis factory to the fabrication yard, despite this being a much smaller distance (as a result of cumulative effect of annual maintenance). The categories that are most sensitive to this change are those where sea vessel operations are important, such as terrestrial acidification, again demonstrating the significance of sea vessel operations to the results of this study. As the sensitivity of the results to location was found to be linear with distance, as illustrated in Fig. 6, adjustment factors are included in Section S3 of the Electronic Supplementary Material to allow the life cycle impacts of the Pelamis to be estimated for other installation locations. There is the potential to reduce the impacts significantly if the offshore distance can be reduced to without significantly compromising the annual energy production.

### Data quality

ISO 14044 (2006) and Astudillo et al. (2017) recommend that the quality of input data to an LCA should be assessed in terms of representativeness, completeness, consistency, reproducibility, uncertainty, data sources and precision. While all input data, assumptions and uncertainty estimates are detailed in the supplementary information, this section summarises the data quality.

#### Representativeness

Geographically, the foreground data was for a scenario of a Pelamis P1 built and installed in Scotland. Background data from ecoinvent used average global materials data and average European manufacturing and transport process data, with additional background data sourced from manufacturers that supply products to Scotland.

Temporally, the scenario was for a device manufactured and installed in 2006 with a lifetime of 20 years and all foreground data gathered in 2006. Background data from ecoinvent v3.3 is valid for 2011 to 2016 so the mismatch in temporal coverage of the two data sets introduces minor uncertainty into the results.

#### Completeness

The foreground data provided by the manufacturer covered all life cycle processes. With the scenario assuming that major steel components were processed at a fabrication yard, the only allowances made for transport of much of the mass of the device are within the generic global market data provided by ecoinvent. As the authors had access to the original calculations by Parker et al. (2007), it was determined that there was no material impact from the decision to exclude small-scale pre-fabricated components. The impacts of some large electrical components and the glass-flake paint were assessed by considering the mass only, while other processes such as sand blasting were modelled based on available manufacturer’s information. Some relevant environmental flows may have been omitted (Electronic Supplementary Material, Fig. S1.2); however, as impacts are dominated by the production of steel used for the main structure, this is expected to have no material impact on the results.

#### Consistency and reproducibility

All background data was ultimately sourced from ecoinvent, with some instances where additional information was needed to link the ecoinvent data with known foreground processes. The supplementary information contains comprehensive information to allow the analysis to be reproduced.

The selected recycling allocation method for the background data was the recycled content method, in order to be consistent with the method chosen for the foreground data. The APOS method is, however, considered to be a more consistent allocation method for attributional LCA, and could be considered preferable for use in studies such as this one (Schrijvers et al. 2016). Furthermore, earlier versions of ecoinvent (including v2, v3 and v3.01) did not include data for the recycled content method (Weidema et al. 2013), and therefore studies based on these datasets will have used the APOS data. In order to examine the impact of selecting recycled content background data, the analysis was re-run with the APOS method and it was found that this changed most impacts by less than 5%. The full results are in the Electronic Supplementary Material, Table S4.1.

#### Data sources and precision

Some epistemic uncertainty may have been introduced by the quality of data gathered by Parker et al. (2007), such as in the derivation of information from fabrication drawings. Also, some background data used to link the available ecoinvent data to the foreground processes was sourced from a single manufacturer. The uncertainty was assessed using the pedigree matrix.

Twenty-seven percent by mass of the substances consumed or emitted do not have characterisation factors in the ReCiPe method. Specifically, 22% was consumption of raw substances which do not fit into the water, metal and fossil depletion categories and 5% was substances emitted to water; their omission may result in slight underestimates of the water impact categories.

## Discussion

This detailed life cycle assessment provides comprehensive information about the environmental impacts of the Pelamis wave energy converter, but the value of this information lies in allowing the Pelamis to be compared with other generation technologies, and in identifying the opportunities for reducing the environmental impacts of future models.

### Comparison with other types of generation

**Fig. 7:**
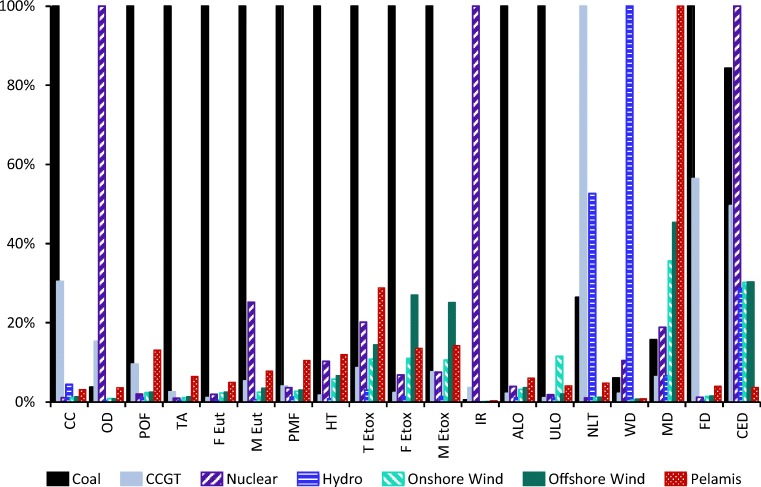
The impacts of the Pelamis in comparison to other types of generation, presented relative to the highest score in each category. Impacts for typical generation technologies  is calculated from processes within ecoinvent v3.3 and detailed in Section S1.5 of the Electronic Supplementary Material (ecoinvent, 2016). Acronyms are defined in Table 5

The Pelamis is intended to be a low-carbon alternative to conventional power generation, but there is also an expectation that it will have much lower environmental impacts across all categories. In order to assess this, the environmental impacts were calculated for a range of different typical technologies from the ecoinvent database (the selected processes are listed in Section S1.5 of the Electronic Supplementary Material). The results are summarised in Fig. 7 and are presented as values relative to the highest impact score in each category. It can be seen that, while the Pelamis does have significantly lower impacts than coal and gas-fired generation (CCGT) in climate change, ionising radiation, natural land transformation, water depletion and fossil and cumulative energy demand, conventional CCGT and nuclear generation perform better than the Pelamis in most other categories. Of all types of generation considered, the Pelamis was found to have the highest impacts in the metal depletion category, and only coal generation performed worse than the Pelamis in a further 7 of the 19 categories.

A comparative uncertainty analysis of Pelamis with each of these other types of generation impacts found that the relationships shown in Fig. 7 generally hold across many impact categories (results in the Electronic Supplementary Material, Table S2.4). The notable exceptions are water depletion across all types of generation, ozone depletion relative to coal and human toxicity relative to nuclear. These uncertainties, therefore, leave the conclusions of this comparison largely unchanged, with the exception of the water depletion category; here the probability that the Pelamis has lower impacts than the other types of generation is only 41–56%.

It should be noted that not all impact categories may be considered equally important; for example, metal depletion may be considered to be of less concern than climate change. The relatively poor performance of the Pelamis in many categories is an interesting result, however, particularly as photochemical oxidant formation and acidification have been significant environmental concerns in recent decades. This highlights the importance of assessing more than just climate change and energy impacts for renewable generators. Furthermore, competing renewable technologies perform better than the Pelamis in most impact categories, although it is very important to remember that they are much more established commercial technologies.

As discussed in Section 4.1, most of the environmental impacts of the Pelamis arise from coke combustion during steel production and diesel combustion for sea vessels; as such, many of the negative effects apparent here are a direct effect of current fossil-based economies. There is, therefore, the potential that significant impact reductions will be made as global infrastructure and technology evolves. The potential for modifying the design of the Pelamis to reduce the environmental impacts of future models is discussed in Section 5.3.

### Comparison with other studies of WECs

Although results of an LCA are only truly comparable with findings from studies based on the same characterisation factors, there is the risk that key results such as carbon and energy impacts might be compared by non-experts with no reference to the methods used. In this study, the climate change and CED of the Pelamis were found to be 35 g CO_2_eq/kWh and 483 kJ/kWh, respectively. These are significantly higher than most other estimates of carbon and energy impacts of WECs, although the climate change impacts compare well with Uihlein’s result for a similar device (2016).

The discrepancy in results between this analysis and that by Parker et al. (2007) is of interest, as both were based on the same case study, but this analysis found the climate change impact and CED to be 53 and 68% higher, respectively. This discrepancy is due to the different data sources and analysis methods applied. Two key sources of variation have been identified. The first of these is that the study by Parker et al. sourced all carbon and energy data from the Inventory of Carbon and Energy (Hammond and Jones, 2008); the method by which embodied energy was calculated in this database is unclear, but the climate change impacts considered only emissions of carbon dioxide. Secondly, the earlier study applied the end-of-life or closed-loop approximation method to the foreground data for calculating the impacts of recycling steel, by assuming that all input steel was primary steel and applying a credit at the disposal stage for the avoided impacts of primary steel (an explanation of this method is given in Schrijvers et al. (2016)).

In order to assess the effect of these methodological choices, the carbon and energy impacts were re-calculated using an approximation of the same recycling allocation method for the foreground data (the recycled content system model was again selected for the background data, to most closely reflect the method applied by Parker et al.). It was assumed that only primary steel was consumed at the manufacturing stage, and a credit was applied for the difference between the impacts of recycled and primary steel at the end-of-life (described further in Section S4 of the Electronic Supplementary Material). This reduced the estimates of climate change and cumulative energy demand significantly, respectively, to 22 and 44% higher than Parker et al. When only CO_2_ emissions were considered, the discrepancy in carbon impacts was reduced to only 16%. This highlights the significance of recycling allocation and impact assessment on this case study. A significant discrepancy still remains which is thought to be due to the information within the ecoinvent database being more comprehensive than that in the Inventory of Carbon and Energy; for example, by including the impacts of capital goods and global market flows. It is important to note that the end-of-life recycling method is no longer recommended for attributional LCA (Schrijvers et al. 2016).

This study also found the sea vessel impacts of installation, maintenance and disposal life cycle stages to be significant across all categories, in contrast to the findings of Uihlein (2016). It is likely that the main reason for this is that the analysis by Uihlein estimated sea vessel requirements to be only 26 h for installation and 100 h/year for maintenance, in contrast to the 1140 h for installation and 130 h/year for maintenance estimated by PWP (which is known to be a very conservative estimate, largely due to the inclusion of sea trials in the installation stage and the case study installation location being very far from port). There is, however, another potential discrepancy in the estimated fuel consumption of the specialist sea vessels: this study used data from PWP for the actual fuel consumption of the sea vessels used, while many LCA studies of offshore renewable energy converters (including that by Uihlein) base their analysis on typical data for large freight ships available within existing datasets. The latter will have a very different fuel consumption than specialist sea vessels; for example, the bulk commodity carriers used by Uihlein to approximate specialist sea vessels have a fuel consumption of 10,000 l/day (assuming fully loaded and travelling at 14 knots) (Thinkstep 2017), while the data from PWP estimated the consumption of the smaller vessels to be 290 to 1710 l/day, depending on vessel type.

While LCA provides a broad set of impact categories, it is by no means a complete categorisation of environmental impacts. The location of WECs offshore means visual impacts are limited and while they undoubtedly occupy areas of sea, the proportions tend to be modest. There has been much ecological research on potential impacts of wave and tidal energy and Bonar et al. (2015) provides an excellent review; this is briefly summarised here. The potential for changes to habitats, particularly of benthic populations (animals that live near to the seabed) arise from the physical changes in wave climate and nearshore currents which influence nutrient levels, as well as disruption from vessel anchoring, trenching for cable installation and permanent anchors for devices. Devices and structures may create artificial reefs which attract biodiversity and the potential for marine reserves due to ‘no fishing’ zones around installations. Electromagnetic fields from power cables have also been an area of concern, and while some fish appear to respond, the risks are considered low. Collision risk with slow moving WECs appears to be quite low, but there is potential for entanglement with moorings, and arrays may be challenging obstacles to navigate. Underwater noise has been a concern, particularly with cetaceans but is mostly associated with increased vessel use and pile driving (not relevant for Pelamis). Finally, there are potential pollution risks from chemical spills during maintenance and leachate from anti-fouling paint. Many of the concerns appear to be minimal but ongoing testing and long-term monitoring will be needed to confirm this (Copping et al. 2014; Leeney et al. 2014).

### Potential for improvement

The results of this comprehensive LCA highlight the life cycle stages with the most significant environmental impacts; this can be used to inform and guide future design developments. Section 4.1 shows that two life cycle stages have high impacts across all categories: the consumption of raw materials, particularly steel and the operation of sea vessels, particularly for maintenance. In order to reduce the environmental impacts of the Pelamis, future design developments should consider reducing the quantity of steel or increasing the recycled content of the steel. As described in Section 4.1, recycled steel has lower impacts across most impact categories with the exception of terrestrial ecotoxicity and ionising radiation, so such a change would reduce most environmental impacts.

The environmental impacts of operating sea vessels are also significant within the life cycle of the Pelamis, and, therefore, are an area to target for improvement. As discussed in Section 4.2, this could be achieved by selecting an installation site nearer to a port, but also any design developments that reduce the requirement for sea vessels, such as the frequency of maintenance operations, will reduce the impacts across most categories.

## Conclusions

This paper presents a detailed life cycle assessment of the first-generation Pelamis wave energy converter, expanding an earlier carbon and energy audit carried out by Parker et al. (2007) to examine a broader range of environmental impacts. Every stage of the device life cycle was considered and all foreground data was sourced from information derived from PWP’s own records by Parker et al. (2007). Background data was sourced from ecoinvent v3.3, and the impacts were calculated with the ReCiPe and CED impact assessment methods (ecoinvent 2016; Hischier et al. 2010; ReCiPe 2016).

The case study analysed was for the production of a single Pelamis P1 device installed at a wave farm 320 km from port off the north-west coast of Scotland. The results of this full LCA confirm that the Pelamis has lower climate change, ionising radiation, natural land transformation, water depletion and fossil and cumulative energy demand impacts when compared to conventional fossil-fuelled power generation. In 8 of the 19 impact categories studied, however, the Pelamis was found to perform worse than most or all other forms of generation, which is of concern for a renewable generator. These negative effects are, however, mostly attributable to the current fossil-based economies, so there is scope for future reductions as global infrastructure and technology evolves.

In the two key impact categories most frequently studied for WECs—climate change and cumulative energy demand—the Pelamis was found to perform reasonably well, with a carbon payback period of 24 months based on current average emissions in Great Britain, and an EROI of 7.3. The poor performance in other impact categories highlights the importance of considering a broad range of environmental impacts in LCA.

Areas with significant potential to reduce the environmental impacts of future Pelamis models were identified: the impacts of the large quantity of steel used to form the main structure of the Pelamis are high, so any reduction in steel mass or increase in recycled content should decrease all environmental impacts; also, the impacts of sea vessel operations are also significant, demonstrating the need to refine the design to reduce maintenance requirements or select an installation location much nearer to port.

## Electronic supplementary material

ESM 1 (PDF 987 kb)
