# Supplementary material for: An LCA of the Pelamis wave energy converter
Source: Int J Life Cycle Assess. 2018 Jul 23;24(1):51–63. doi: 10.1007/s11367-018-1504-2 (PMC6383585; doi:10.1007/s11367-018-1504-2)
Supplement: Supplementary file 1 — (PDF 987 kb) [file 11367_2018_1504_MOESM1_ESM.pdf]

Supplementary Material to:

An LCA of the Pelamis Wave Energy Converter

R. Camilla Thomson[1], John P. Chick and Gareth P. Harrison

School of Engineering, University of Edinburgh, Edinburgh, EH9 3DW, UK

Published in the **International Journal of Life Cycle Assessment**

[1] Corresponding author: c.thomson@ed.ac.uk

S1 Input Data

S1.1 Key parameters

The analysis presented in this paper is an LCA of a single case-study manufacturing and installation scenario of the Pelamis Wave Energy Converter (WEC). In order to facilitate the use of this analysis in any “meta-models” of wave energy, it has been suggested by Astudillo et al. (2017) that a number of key parameters should be clearly reported. These are all detailed in the main text, but for clarity are summarised below.

Location	Off the north-west coast of Scotland
Technological maturity	First production machines; i.e. ascent stage
Installation year	2006
Period of validity	2006-2010[2]
Capacity	750kW
Operating lifetime	20 years
Capacity factor	45%
Annual energy production	2.97GWh
Technology type	Attenuator-type floating oscillating body system wave energy converter
Data type	Empirical from cradle to completed installation,<br>Theoretical for maintenance, decommissioning and disposal
Plant production and decommissioning	Included
Characterization factors	ReCiPe midpoint method, hierarchist version with European normalisation,<br>Cumulative energy demand
Mass	1040 tonnes

S1.2 Data from manufacturer and detailed life cycle

The process of calculating the Life Cycle Inventory is described in Figure S1.1. Table S1.1, Table S1.2, Table S1.3 and Table S1.4 summarise the input data derived from information provided by Pelamis Wave Power Ltd (PWP), along with the selected process from ecoinvent v3.3 and the uncertainty indicator scores. The last refer to ratings used to estimate the uncertainty according to the same pedigree matrix used in the ecoinvent database, and described in Section 3.5 of the main report (Weidema, et al., 2013). Figure S1.2 describes the life cycle flows included/excluded from the study.

[2] Device design was superceded in 2010, but no data was gathered for an LCA of the later version of the machine before the manufacturer went into administration in 2014.

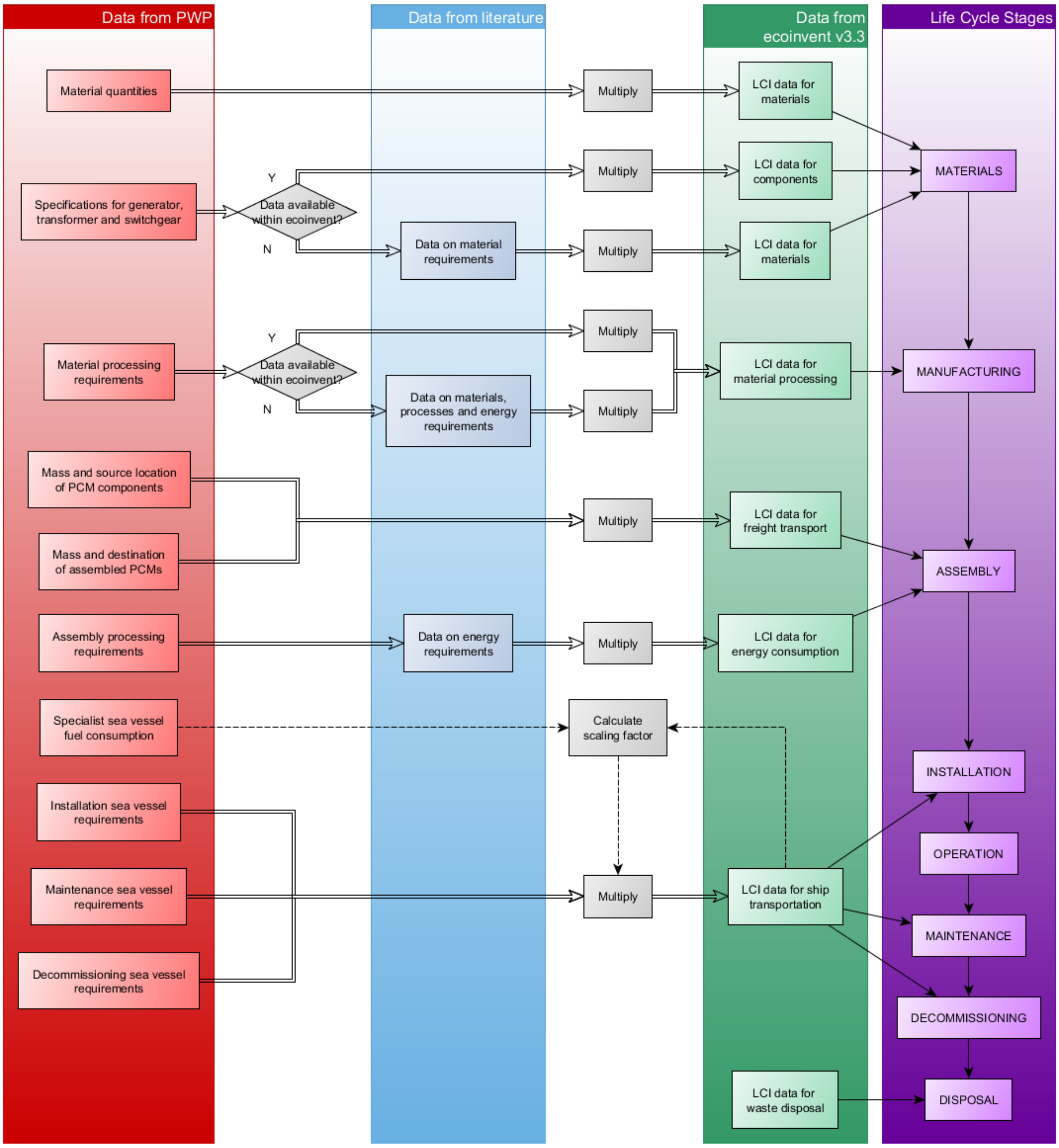

Figure S1.1 - Process of calculating life cycle inventory data from input data provided by Pelamis Wave Power Ltd.

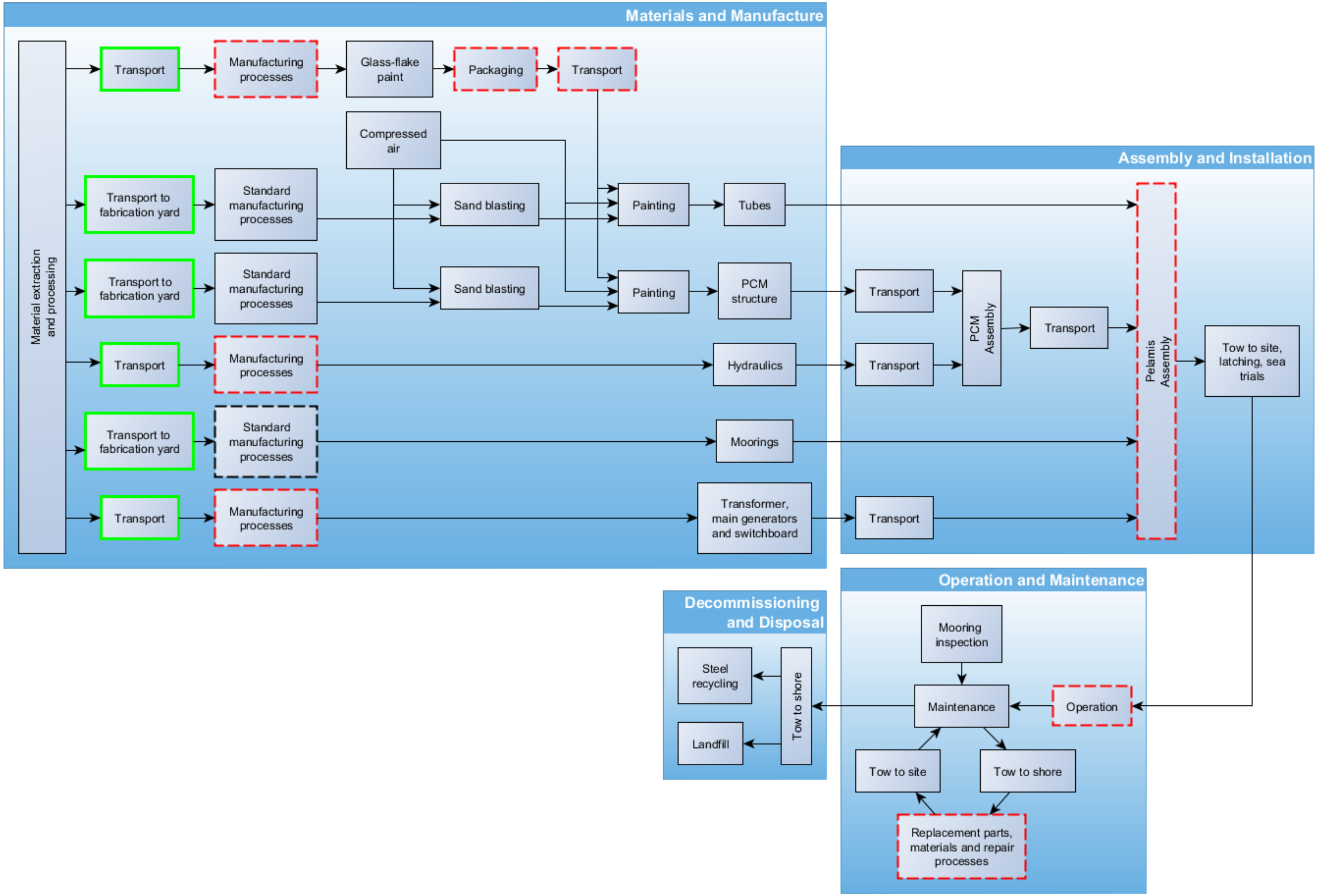

Figure S1.2 - Flow chart describing the system evaluated. Processes framed in green are assumed to be included in the ecoinvent data. Processes framed in a red dashed line are excluded from the analysis

Data from Manufacturer	Quantity	Unit	SD[2]	Uncertainty Indicators	Selected Inventory Process
**General Data**					
Annual energy production	2.97	GWh	1.050	2, 1, 1, 1, 1	
Design life	20	years	1.196	4, 1, 1, 1, 1	
Recycling rate	90	%	1.204	4, 1, 1, 2, 2	
**Stock Material**					
Steel - cast	221982	kg	1.058	1, 1, 3, 1, 1	Steel, low-alloyed {GLO}| market for
Steel - plate	345901	kg	1.058	1, 1, 3, 1, 1	Steel, low-alloyed, hot rolled {GLO}| market for
Sand	475722	kg	1.058	1, 1, 3, 1, 1	Sand {GLO}| market for
Stainless steel	550	kg	1.058	1, 1, 3, 1, 1	Steel, chromium steel 18/8 {GLO}| market for
Nylon 6	416	kg	1.058	1, 1, 3, 1, 1	Nylon 6 {GLO}| market for
Polyurethane[3]	3.5	m^3	1.058	1, 1, 3, 1, 1	Polyurethane, rigid foam {GLO}| market for
Glass reinforced plastic (GRP)	90	kg	1.058	1, 1, 3, 1, 1	Glass fibre reinforced plastic, polyamide, injection moulded {GLO}| market for
PVC pipe	55	kg	1.058	1, 1, 3, 1, 1	Polyvinylchloride, suspension polymerised {GLO}| market for[4]
**Manufacturing Processes**					
Drawing of steel pipes	6383	kg	1.058	1, 1, 3, 1, 1	Drawing of pipe, steel {GLO}| market for
Drawing of steel wire	460	kg	1.058	1, 1, 3, 1, 1	Wire drawing, steel {GLO}| market for
Extruding plastic pipes	55	kg	1.058	1, 1, 3, 1, 1	Extrusion, plastic pipes {GLO}| market for
Machining	53924	cm^3	1.206	1, 1, 3, 1, 3	Steel removed by milling, average {RER}| steel milling, average[5]
Welding	1995	m	1.058	1, 1, 3, 1, 1	Welding, arc, steel {RER}| processing
Flame cutting	41	m^2	1.058	1, 1, 3, 1, 1	Approximated from gas welding. Detailed in Table S1.5.
Abrasive blasting	2025	m^2	1.058	1, 1, 3, 1, 1	Derived from published information. Detailed in Table S1.5Table S1.9.
Painting	2025	m^2	1.058	1, 1, 3, 1, 1	Derived from published information. Detailed in Table S1.5.

Table S1.1 - Input data for materials and manufacturing provided by PWP, with details of corresponding inventory processes and uncertainty indicator scores.

[3] Density 110 kg/m^3 from Trelleborg (2009).

[4] With pipe extrusion, as detailed under Manufacturing Processes.

[5] The PWP data for machining includes all small-scale precision removal of material, such as milling, grinding and drilling.

Data from Manufacturer	Quantity	Unit	SD[2]	Uncertainty Indicators	Selected Inventory Process
**Pre-fabricated components**					
Main Generator, 175kW	1	unit	1.119	1, 1, 3, 2, 3	Generator, 200kW electrical {GLO}| market for[6]
MV Switchgear and TSG Control Panel	1	unit			Derived from published information. Detailed in Table S1.9.
Transformer, 315kVA, 11kV	1	unit			Derived from published information. Detailed in Table S1.9.

Table S1.2 - Information on electrical components provided by PWP, with details of corresponding inventory processes and uncertainty indicator scores.

[6] Generator for a gas cogeneration unit.

Data from Manufacturer	Quantity	Unit	SD[2]	Uncertainty Indicators	Selected Inventory Process
**Transport**					
Distance uncertainty - city of origin known			2.003	2, 1, 1, 1, 1	
Distance uncertainty - country of origin known			2.011	3, 1, 1, 1, 1	
**Small lorry**					**Transport, freight, lorry 3.5-7.5 metric ton, EURO3 {RER}[7]**
From UK to Methil - estimated	0.48	kg	1.221	4, 1, 3, 1, 1	
From UK to Methil - manufacturer's data	1.35	kg	1.094	1, 1, 3, 1, 1	
**Large lorry**					**Transport, freight, lorry 16-32 metric ton, EURO3 {RER}[7]**
From Scotland to Methil - estimated	0.06	t	1.221	4, 1, 3, 1, 1	
From Glasgow to Methil - manufacturer's data	0.18	t	1.094	1, 1, 3, 1, 1	
From Stonehaven to Methil - manufacturer's data	69.62	t	1.094	1, 1, 3, 1, 1	
From Nottingham to Methil - manufacturer's data	17.40	t	1.094	1, 1, 3, 1, 1	
From UK to Methil - estimated	7.86	t	1.221	4, 1, 3, 1, 1	
From Wales to Methil - manufacturer's data	9.00	t	1.094	1, 1, 3, 1, 1	
From Methil to Stornoway	106.55[8]	t			
**Sea freight**					**Transport, freight, sea, transoceanic ship {GLO}| market for**
From China to Methil - estimated	0.30	kg	1.221	4, 1, 3, 1, 1	
From Holland to Methil - estimated	0.30	kg	1.221	4, 1, 3, 1, 1	

Table S1.3 - Input data for transport of components for power conversion modules provided by PWP or estimated, with details of corresponding inventory processes and uncertainty indicator scores.

[7] Assuming EURO3 standard, as this has the highest emissions and is therefore the most conservative
[8] Sum of all component estimates

Data from Manufacturer	Quantity	Unit	SD^2	Uncertainty	Selected Inventory Process
**Assembly processes**					
60T crane	120	hrs	1.107	2, 1, 3, 1, 1	Derived from published information. Detailed in Table S1.9.
Fork-lift truck	14.1	hrs	1.107	2, 1, 3, 1, 1	Derived from published information. Detailed in Table S1.9.
**Installation processes**					
Barge	11.84	days	1.094	3, 1, 1, 1, 1	Derived from data from Pelamis Wave Power. Detailed in Table S1.9.
Multicat	24	days	1.094	3, 1, 1, 1, 1	Derived from data from Pelamis Wave Power. Detailed in Table S1.9.
Tug	11.83	days	1.094	3, 1, 1, 1, 1	Derived from data from Pelamis Wave Power. Detailed in Table S1.9.
**Maintenance processes**	**Per year**				
Tug	4.00	days	1.094	3, 1, 1, 1, 1	Derived from data from Pelamis Wave Power. Detailed in Table S1.9.
Inspection vessel	1.33	days	1.094	3, 1, 1, 1, 1	Derived from data from Pelamis Wave Power. Detailed in Table S1.9.
**Decommissioning processes**					
Barge	2.50	days	1.094	3, 1, 1, 1, 1	Derived from data from Pelamis Wave Power. Detailed in Table S1.9.
Multicat	8.50	days	1.094	3, 1, 1, 1, 1	Derived from data from Pelamis Wave Power. Detailed in Table S1.9.
Tug	2.50	days	1.094	3, 1, 1, 1, 1	Derived from data from Pelamis Wave Power. Detailed in Table S1.9.

Table S1.4 - Input data for assembly and specialist sea vessel processes provided by PWP, with corresponding uncertainty indicator scores.

S1.3 Process approximations

The ecoinvent database does not contain detailed inventory information for some specialist materials, manufacturing processes and sea vessel operations. In order to assess the resource use and pollutant emissions associated with these, data on material quantities and fuel consumption were sourced elsewhere, and new processes were built using inventory data from ecoinvent. The selected materials, quantities and associated uncertainty are given in Table S1.5, Table S1.6, Table S1.7, Table S1.8 and Table S1.9.

S1.4 Waste disposal processes

Table S1.10 details the waste disposal processes selected from the ecoinvent dataset for each of the principal materials within the analysis.

S1.5 Alternative electricity generation

In order to compare the environmental impacts of the Pelamis with those from other types of power generation, selected average electricity generation data from a number of key energy sources was analysed with the ReCiPe Midpoint Hierarchist and Cumulative Energy Demand impact assessment methods. The processes selected from the ecoinvent database (v3.3) are detailed in Table S1.11.

Process or material	Quantity	Unit	SD[2]	Uncertainty	Selected Inventory Process/emission
**Flame cutting**	**per m^2**				
Gas welding	50	m	1.288	4, 1, 1, 1, 3	Welding, gas, steel {RER}| processing
**Sand blasting**	**per m^2**				
Abrasive for jet blasting[9]	10	kg	1.237	2, 4, 1, 3, 3	Silica sand {GLO}| market for
Compressed air supply for jet blasting[10]	5.8	m^3	1.258	2, 4, 1, 5, 3	Compressed air, 800 kPa gauge {RER}| <30kW, average generation
Particulate emissions[11]	1.35E-05	kg	2.050	1, 3, 2, 3, 3	Particulates, > 2.5 um, and < 10um
**Painting, glass flake paint[12]**	**per m^2**				
Epoxy paint primer/topcoat[13]	0.808	kg	1.196	4, 1, 1, 1, 1	Detailed below.
Glass flake paint 35851[13]	1.23	kg	1.196	4, 1, 1, 1, 1	Detailed below.
Compressed air supply for painting[14]	12.8	m^3	1.131	2, 4, 1, 1, 2	Compressed air, 700 kPa gauge {RER}| >30kW, average generation
**Glass flake paint 35851[15]**	**per kg**				
Curing Agent 97652	0.034	kg	1.107	1, 4, 1, 1, 1	Detailed in Table S1.6.
Base 35858	0.183	kg	1.107	1, 4, 1, 1, 1	Detailed in Table S1.6.
Glass flakes	0.781	kg	1.107	1, 4, 1, 1, 1	Flat glass, uncoated {GLO}| market for
**Epoxy paint primer/topcoat[16]**	**per kg**				
Curing Agent 97652	0.156	kg	1.107	1, 4, 1, 1, 1	Detailed in Table S1.6.
Base 35858	0.842	kg	1.107	1, 4, 1, 1, 1	Detailed in Table S1.6.

Table S1.5 - Details of new processes created from data within ecoinvent for manufacturing processes and glass-flake paint, with corresponding uncertainty indicators

---

[9] From Jiven et al. (2004).

[10] Quantity derived from Axxiom (2008). Pressure from Kalpakjian et al. (2008). Compressed air sourced locally, so European average data selected.

[11] From data for abrasive blasting of aluminium in Classen et al. (2009).

[12] The paint is applied with an airless spray at 250 bar, providing a coverage of 3.9 m^2/l with a thickness of 200µm (Hempel, 2007).

[13] Parker et al. (2007) estimated an overall 1mm paint thickness requiring a base coat of primer, three layers of paint and a topcoat.

[14] The paint application process was approximated from manufacturer's data for an airless spray pump (Graco, 2010), powered by 200 m^3/min of compressed air to provide paint coverage of 12 l/min.

[15] From Hempel (2007)

[16] Assumed to be the same as the glass flake paint, without the glass flakes.

Process or material	Quantity	Unit	Uncertainty[17]	Selected Inventory Process
**Curing Agent 97652[18]**	**per kg**			
Xylene	0.1625	kg	0.125 - 0.2	Xylene {GLO}| market for
n-butanol	0.085	kg	0.07 - 0.1	1-butanol {GLO}| market for
p-tert-butylphenol	0.075	kg	0.05 - 0.1	Phenol {GLO}| market for
m-xylylene-diamine	0.04	kg	0.03 - 0.05	Meta-phenylene diamine {GLO}| market for
Ethanol	0.03	kg	0.01 - 0.05	Ethanol, without water, in 99.7% solution state, from ethylene {GLO}| market for
Ethylbenzene	0.05	kg	0.03 - 0.07	Ethyl benzene {GLO}| market for
2,2,4- and 2,4,4-trimethylhexamethylene diamine	0.0175	kg	0.01 - 0.025	Ethylenediamine {GLO}| market for
2,4,6-tris(dimethylaminomethyl)phenol	0.02	kg	0.01 - 0.03	O-aminophenol {GLO}| market for
3-(2-aminoethylamino)propyltrimethoxysilane	0.00625	kg	0.0025 - 0.01	Ethylenediamine {GLO}| market for
Remainder	0.51375	kg	0.365 - 0.6625	Epoxy resin, liquid {GLO}| market for
**Base 35858[19]**	**per kg**			
bisphenol A-(epichlorhydrin) epoxy resin MW =< 700	0.15	kg	0.05 - 0.25	Epoxy resin, liquid {GLO}| market for
middle molecular epoxy resin MW 700-1200	0.075	kg	0.05 - 0.1	Epoxy resin, liquid {GLO}| market for
Xylene	0.075	kg	0.05 - 0.1	Xylene {GLO}| market for
n-butanol	0.04	kg	0.03 - 0.05	1-butanol {GLO}| market for
Ethylbenzene	0.02	kg	0.01 - 0.03	Ethyl benzene {GLO}| market for
solvent naphtha (petroleum), light arom.	0.0175	kg	0.01 - 0.025	Naphtha {RER}| market for
alpha'-(1,3-xylenediyl)bis(12-hydroxyoctadecanamide)	0.02	kg	0.01 - 0.03	Dimethenamide {GLO}| market for
Remainder	0.6025	kg	0.415 - 0.79	Epoxy resin, liquid {GLO}| market for

Table S1.6 - Details of materials within glass-flake paint, selected ecoinvent data and corresponding uncertainty indicators

[17] Uncertainty ranges taken from material data sheets
[18] (Hempel, 2010b)
[19] (Hempel, 2010a)

Process or material	Quantity	Unit	SD[2]	Uncertainty Indicators	Selected Inventory Process
**MV Switchgear and TSG Control Panel**	**per unit**				
MV switch-disconnector cubicle[20]	2				Detailed below
SF6 MV circuit breaker	1				Detailed in Table S1.8.
**MV switch-disconnector cubicle[21]**	**per unit**				
Steel	89.7	kg	1.231	1, 4, 2, 2, 3	Steel, low-alloyed, hot rolled {GLO}| market for
Stainless steel	6	kg	1.231	1, 4, 2, 2, 3	Steel, chromium steel 18/8, hot rolled {GLO}| market for
Copper	7.6	kg	1.231	1, 4, 2, 2, 3	Copper {GLO}| market for
Brass	0.4	kg	1.231	1, 4, 2, 2, 3	Brass {RoW}| market for brass
Polycarbonate	0.9	kg	1.231	1, 4, 2, 2, 3	Polycarbonate {GLO}| market for
EPDM	0.7	kg	1.231	1, 4, 2, 2, 3	Synthetic rubber {GLO}| market for
Polypropylene	0.1	kg	1.231	1, 4, 2, 2, 3	Polypropylene, granulate {GLO}| market for
Polyester	0.1	kg	1.231	1, 4, 2, 2, 3	Polyester resin, unsaturated {GLO}| market for
Glass	0.1	kg	1.231	1, 4, 2, 2, 3	Flat glass, uncoated {GLO}| market for
Epoxy	22.6	kg	1.231	1, 4, 2, 2, 3	Epoxy resin, liquid {GLO}| market for
Sulphur hexafluoride	0.2	kg	1.231	1, 4, 2, 2, 3	Sulfur hexafluoride, liquid {GLO}| market for
Zinc	0.5	kg	1.231	1, 4, 2, 2, 3	Zinc {GLO}| market for
Aluminium	1.1	kg	1.231	1, 4, 2, 2, 3	Aluminium, wrought alloy {GLO}| market for
Paint	0.8	kg	1.231	1, 4, 2, 2, 3	Alkyd paint, white, without water, in 60% solution state {GLO}| market for

Table S1.7 - Details of materials in MV switch-disconnector cubicle, selected ecoinvent data and corresponding uncertainty indicators

[20] Second cubicle representing TSG control panel
[21] (ABB, 2010)

Process or material	Quantity	Unit	SD[2]	Uncertainty Indicators	Selected Inventory Process
**SF6 MV circuit breaker[22]**	**per unit**				
Steel	55.043	g	1.231	1, 4, 2, 2, 3	Steel, low-alloyed {GLO}| market for
Stainless steel	1.332	g	1.231	1, 4, 2, 2, 3	Steel, chromium steel 18/8, hot rolled {GLO}| market for
Aluminium	899	g	1.231	1, 4, 2, 2, 3	Aluminium, wrought alloy {GLO}| market for
Alumina	378	g	1.231	1, 4, 2, 2, 3	Aluminium oxide {GLO}| market for
Copper	16.736	g	1.231	1, 4, 2, 2, 3	Copper {GLO}| market for
Copper tungsten 20	315	g	1.231	1, 4, 2, 2, 3	Copper {GLO}| market for
Polyamide 11	15	g	1.231	1, 4, 2, 2, 3	Nylon 6 {GLO}| market for
Polyamide 66	183	g	1.231	1, 4, 2, 2, 3	Nylon 6-6 {GLO}| market for
Polycarbonate	140	g	1.231	1, 4, 2, 2, 3	Polycarbonate {GLO}| market for
Polycarbonate+FB30	61	g	1.231	1, 4, 2, 2, 3	Polycarbonate {GLO}| market for
PVC	8	g	1.231	1, 4, 2, 2, 3	Polyvinylchloride, suspension polymerised {GLO}| market for
Bronze	9	g	1.231	1, 4, 2, 2, 3	Bronze {GLO}| market for
PTFE	227	g	1.231	1, 4, 2, 2, 3	Tetrafluoroethylene {GLO}| market for
Epoxy Resin	23.751	g	1.231	1, 4, 2, 2, 3	Epoxy resin, liquid {GLO}| market for
Epoxy Resin Fe10	844	g	1.231	1, 4, 2, 2, 3	Epoxy resin, liquid {GLO}| market for
SF6	282	g	1.231	1, 4, 2, 2, 3	Sulfur hexafluoride, liquid {GLO}| market for
Brass	198	g	1.231	1, 4, 2, 2, 3	Brass {RoW}| market for brass
**Transformer, 315kVA, 11kV[23]**				**per unit**	
Core Steel	533	kg	1.231	1, 4, 2, 2, 3	Steel, low-alloyed {GLO}| market for
Transformer oil	340	kg	1.231	1, 4, 2, 2, 3	Vegetable oil methyl ester {GLO}| market for
Steel tank	324	kg	1.231	1, 4, 2, 2, 3	Steel, low-alloyed, hot rolled {GLO}| market for
Aluminium wire	113.51	kg	1.231	1, 4, 2, 2, 3	Aluminium, primary, cast alloy slab {GLO}| market for
Aluminium sheet	86.3	kg	1.231	1, 4, 2, 2, 3	Aluminium, wrought alloy {GLO}| market for
Transformer insulation material	59.9	kg	1.231	1, 4, 2, 2, 3	Kraft paper, unbleached {GLO}| market for
Porcelain	11	kg	1.231	1, 4, 2, 2, 3	Sanitary ceramics {GLO}| market for

Table S1.8 - Details of materials in SF6 breaker and transformer, selected ecoinvent data and corresponding uncertainty indicators

[22] (ABB, 2001)
[23] (ABB, 2007)

Process or material	Quantity	Unit	SD[2]	Uncertainty	Selected Inventory Process
**60T Crane[24]**	**per hour**				
Electricity	18	kWh	1.568	4, 4, 1, 2, 4	Electricity, low voltage {GB}| market for
**Fork lift truck[25]**	**per hour**				
Diesel	2.55	kg	1.511	1, 4, 1, 2, 4	Diesel, burned in building machine {GLO}| processing
**Sea vessels[26]**	**per day**				
Barge	113000	tkm	2.057	1, 1, 3, 1, 3	Transport, freight, sea, transoceanic ship {GLO}| market for
Multicat	5780000	tkm	2.057	1, 1, 3, 1, 3	Transport, freight, sea, transoceanic ship {GLO}| market for
Tug	663000	tkm	2.057	1, 1, 3, 1, 3	Transport, freight, sea, transoceanic ship {GLO}| market for
Inspection vessel	194000	tkm	2.057	1, 1, 3, 1, 3	Transport, freight, sea, transoceanic ship {GLO}| market for

Table S1.9 - Details of new processes created from data within ecoinvent for manufacturing processes and sea vessel operations, with corresponding uncertainty indicators

[24] (SWF, 2011)
[25] (Caterpillar, 2011)
[26] Scaled to match fuel consumption provided by PWP

Waste Material	Selected Inventory Process
Steel	Scrap steel {Europe without Switzerland}| treatment of scrap steel, inert material landfill
Aluminium	Waste aluminium {RoW}| treatment of, sanitary landfill
Other metals	Scrap steel {Europe without Switzerland}| treatment of scrap steel, inert material landfill
PVC	Waste polyvinylchloride {Europe without Switzerland}| treatment of waste polyvinylchloride, sanitary landfill
Other plastics	Waste plastic, mixture {Europe without Switzerland}| treatment of waste plastic, mixture, sanitary landfill
Other materials	Inert waste, for final disposal {RoW}| treatment of inert waste, inert material landfill

Table S1.10 - Waste processing datasets selected from ecoinvent.

Type of generation	Process Name
Coal	Electricity, high voltage {GB}| electricity production, hard coal
Gas (CCGT)	Electricity, high voltage {GB}| electricity production, natural gas, combined cycle power plant
Nuclear	Electricity, high voltage {GB}| electricity production, nuclear, pressure water reactor[27]
Hydro	Electricity, high voltage {RoW}| electricity production, hydro, reservoir, non-alpine region
Onshore Wind	Electricity, high voltage {GB}| electricity production, wind, 1-3MW turbine, onshore
Offshore Wind	Electricity, high voltage {GB}| electricity production, wind, 1-3MW turbine, offshore

Table S1.11 - Source data for comparison with other types of generation

[27] Only one nuclear power station in the UK is a pressurised water reactor. The remainder are advanced gas-cooled reactors, but as this is an old technology that is rarely used elsewhere, data for it is not included in ecoinvent v3.3.

S2 Additional Numerical Results

This section contains additional results not presented in the main article. Table S2.1 gives the breakdown of cumulative energy demand results for each primary energy carrier.

Category	Value (kJ/kWh)
Non-renewable, fossil	445
Non-renewable, nuclear	23
Non-renewable, biomass	0.062
Renewable, biomass	6.4
Renewable, wind, solar, geothermal	1.7
Renewable, water	17
**TOTAL**	493

Table S2.1 - Breakdown of cumulative energy demand

The results of the uncertainty analysis are shown graphically in the paper, but for completeness, the numerical results are given in Table S2.2. Similarly, complete results of the sensitivity analysis are summarised in Table S2.3.

Impact Category	Mean	SD	Median	2.5%	97.5%	Unit
Climate change	35	7	35	25	51	g CO_2 eq/kWh
Ozone depletion	3.7	1.8	3.3	1.5	8.5	µg CFC-11 eq/kWh
Photochemical oxidant formation	331	95	315	190	553	mg PM10 eq/kWh
Terrestrial acidification	410	113	395	244	670	mg NMVOC/kWh
Freshwater eutrophication	21	11	19	10	46	mg SO_2 eq/kWh
Marine eutrophication	14	4	13	9	22	mg P eq/kWh
Particulate matter formation	187	41	183	121	279	g 1,4-DB eq/kWh
Human toxicity	33	22	28	14	79	mg N eq/kWh
Terrestrial ecotoxicity	4.3	1.0	4.1	2.9	6.8	mg 1,4-DB eq/kWh
Freshwater ecotoxicity	930	483	814	429	2191	mg 1,4-DB eq/kWh
Marine ecotoxicity	948	455	840	471	2036	mg 1,4-DB eq/kWh
Ionising radiation	2.4	1.7	2.0	0.8	6.4	Bq ^{235}U eq/kWh
Agricultural land occupation	920	286	865	538	1598	mm^2a/kWh
Urban land occupation	399	94	385	261	608	mm^2a/kWh
Natural land transformation	8.5	5.5	7.6	0.2	21.1	mm^2/kWh
Water depletion	-250	37208	4387	-86649	60055	cm^3/kWh
Metal depletion	26	6	26	17	40	g Fe eq/kWh
Fossil depletion	10	3	10	6.0	16	g oil eq/kWh
Energy	494	121	474	317	793	kJ/kWh

Table S2.2 - Complete results of uncertainty analysis

		Capacity Factor		Distance offshore (km)		Distance from Pelamis plant (km)		Design life (years)	
		25%	55%	20	320	213	633	10	30
**Climate change**	g CO_2 eq/kWh	63	29	24	35	35	35	60	27
**Ozone depletion**	µg CFC-11 eq/kWh	6.8	3.1	2.0	3.7	3.7	3.7	5.89	3.02
**Photochemical oxidant formation**	mg PM10 eq/kWh	588	267	163	325	325	326	505	265
**Terrestrial acidification**	mg NMVOC/kWh	730	332	190	404	403	404	616	333
**Freshwater eutrophication**	mg SO_2 eq/kWh	38	17	20	21	21	21	41	14
**Marine eutrophication**	mg P eq/kWh	25	11	8	14	14	14	22	11
**Particulate matter formation**	g 1,4-DB eq/kWh	333	151	114	184	184	184	306	144
**Human toxicity**	mg N eq/kWh	60	27	32	33	33	33	65	22
**Terrestrial ecotoxicity**	mg 1,4-DB eq/kWh	7.7	3.5	3.8	4.2	4.2	4.3	8.1	3.0
**Freshwater ecotoxicity**	mg 1,4-DB eq/kWh	1638	745	867	906	906	906	1777	615
**Marine ecotoxicity**	mg 1,4-DB eq/kWh	1671	760	862	924	924	925	1793	635
**Ionising radiation**	Bq ^{235}U eq/kWh	4.4	2.0	1.4	2.4	2.4	2.4	4.0	1.9
**Agricultural land occupation**	mm^2a/kWh	1654	752	770	915	914	916	1700	653
**Urban land occupation**	mm^2a/kWh	712	323	334	393	390	397	734	280
**Natural land transformation**	mm^2/kWh	15.3	7.0	4.5	8.5	8.4	8.5	13.4	6.8
**Water depletion**	cm^3/kWh	436	198	208	241	241	242	453	171
**Metal depletion**	g Fe eq/kWh	47	21	26	26	26	26	52	17
**Fossil depletion**	g oil eq/kWh	18.1	8.2	6.4	10.0	10.0	10.0	16.8	7.8
**Energy**	kJ/kWh	892	405	323	493	492	494	833	380

Table S2.3 - Sensitivity analysis results. Highest values for each impact category are highlighted in orange, and lowest values in green.

Impact Category	Probability (%) that impacts of Pelamis are less than:					
	Coal	CCGT	Nuclear	Hydro	Onshore wind	Offshore wind
Climate change	100	100	0.0	86.6	1.3	0.0
Ozone depletion	**50.9**	99.8	100	0.0	0.1	0.0
Photochemical oxidant formation	100	23.3	0.1	0.0	0.0	0.0
Terrestrial acidification	100	1.8	0.0	0.0	0.0	0.0
Freshwater eutrophication	99.9	0.0	1.7	0.0	3.3	0.8
Marine eutrophication	100	17.6	98.5	0.0	0.8	0.1
Particulate matter formation	100	1.2	0.8	0.0	0.1	0.0
Human toxicity	98.5	0.0	**32.6**	0.0	4.9	2.7
Terrestrial ecotoxicity	99.8	0.0	19.2	0.0	1.6	0.3
Freshwater ecotoxicity	99.6	0.1	8.0	0.0	29.0	98.3
Marine ecotoxicity	99.6	11.6	9.4	0.0	20.4	98.0
Ionising radiation	81.1	100	100	0.0	1.3	0.0
Agricultural land occupation	100	0.0	6.1	0.0	5.9	1.1
Urban land occupation	100	0.0	1.8	0.0	98.5	0.0
Natural land transformation	76.4	99.9	0.7	99.9	2.0	0.9
Water depletion	**50.8**	**41.6**	**50.9**	**56.5**	**45.1**	**44.4**
Metal depletion	0.0	0.0	0.0	0.0	1.4	0.0
Fossil depletion	100	100	0.0	0.0	1.6	0.0
Energy	100	100	100	100	100	100

Table S2.4 - Results of comparative uncertainty analysis of Pelamis with other types of generation. Values between 30 and 70% are highlighted, as these show a significant probability that the impacts of the Pelamis relative to the given type of generation may be reversed.

S3 Locational Adjustment Factors

The normalised impact potentials can be estimated for any given installation location, using the following equation:

$$E = \frac{\left(a + b \cdot l_{steel} + c \cdot l_{offshore}\right)}{20W}$$

where:

E	=	Embodied impacts per kWh
l_{steel}	=	Distance from Pelamis plant to steel fabrication yard (km)
$l_{offshore}$	=	Distance from dockyard to installation site (km)
W	=	Annual energy output (kWh)
a, b and c	=	Constants for each impact category (given in Table S3.1)

Note that this formula is a simplification of the results of this analysis, and cannot be used to determine the effect of a change in other factors. Furthermore, this model has been developed for an installation scenario in the UK, and therefore installation in other countries may not have the same impacts.

Impact Potential	*a*	*b*	*c*	
**Climate change**	$1.37\text{x}10^{9}$	$1.73\text{x}10^{4}$	$2.18\text{x}10^{6}$	g CO_2 eq
**Ozone depletion**	$1.09\text{x}10^{8}$	$3.29\text{x}10^{3}$	$3.49\text{x}10^{5}$	µg CFC-11 eq
**Terrestrial acidification**	$1.04\text{x}10^{10}$	$9.15\text{x}10^{4}$	$4.23\text{x}10^{7}$	mg SO_2 eq
**Freshwater eutrophication**	$1.16\text{x}10^{9}$	$1.26\text{x}10^{3}$	$2.88\text{x}10^{5}$	mg P eq
**Marine eutrophication**	$4.30\text{x}10^{8}$	$5.07\text{x}10^{3}$	$1.17\text{x}10^{6}$	mg N eq
**Human toxicity**	$1.89\text{x}10^{9}$	$5.14\text{x}10^{3}$	$2.44\text{x}10^{5}$	g 1,4-DB eq
**Photochemical oxidant formation**	$8.97\text{x}10^{9}$	$1.40\text{x}10^{5}$	$3.22\text{x}10^{7}$	mg NMVOC
**Particulate matter formation**	$6.48\text{x}10^{9}$	$4.62\text{x}10^{4}$	$1.39\text{x}10^{7}$	mg PM10 eq
**Terrestrial ecotoxicity**	$2.21\text{x}10^{8}$	$7.86\text{x}10^{3}$	$8.77\text{x}10^{4}$	mg 1,4-DB eq
**Freshwater ecotoxicity**	$5.13\text{x}10^{10}$	$7.62\text{x}10^{4}$	$7.66\text{x}10^{6}$	mg 1,4-DB eq
**Marine ecotoxicity**	$5.09\text{x}10^{10}$	$1.19\text{x}10^{5}$	$1.23\text{x}10^{7}$	mg 1,4-DB eq
**Ionising radiation**	$8.14\text{x}10^{7}$	$1.51\text{x}10^{3}$	$1.92\text{x}10^{5}$	Bq ^{235}U eq
**Agricultural land occupation**	$4.51\text{x}10^{10}$	$2.10\text{x}10^{5}$	$2.86\text{x}10^{7}$	mm^2a
**Urban land occupation**	$1.92\text{x}10^{10}$	$9.50\text{x}10^{5}$	$1.17\text{x}10^{7}$	mm^2a
**Natural land transformation**	$2.49\text{x}10^{8}$	$6.99\text{x}10^{3}$	$7.82\text{x}10^{5}$	mm^2
**Water depletion**	$1.22\text{x}10^{10}$	$5.04\text{x}10^{4}$	$6.65\text{x}10^{6}$	cm^3
**Metal depletion**	$1.53\text{x}10^{9}$	$6.04\text{x}10^{2}$	$5.12\text{x}10^{4}$	g Fe eq
**Fossil depletion**	$3.64\text{x}10^{8}$	$6.35\text{x}10^{3}$	$7.17\text{x}10^{5}$	g oil eq
**Energy**	$1.84\text{x}10^{10}$	$2.91\text{x}10^{5}$	$3.38\text{x}10^{7}$	kJ

Table S3.1 - Constants for estimating the environmental impacts at alternative locations

S4 Recycling allocation

S4.1 Comparing recycled content method with APOS

Ecoinvent v3.3 includes data for two different allocation methods for attributional LCA: the recycled content method and the “allocation at the point of substitution” (APOS) method. In this study the former was chosen in order to enable consistency in application for foreground recycling processes. The latter is, however, considered by some to be the better approach for more consistent allocation (Schrijvers, et al., 2016). It is also the only available method in earlier versions of the ecoinvent dataset (v3 and v3.01) so will have been applied in other studies that also employ the recycled content method for the foreground data. The analysis was, therefore, re-run with the APOS approach applied to background processes, and results are given in Table S4.1.

S4.2 Approximating the end-of-life recycling method

Section 5.2 of the main article describes how the analysis was re-run using an approximation of the end-of-life method for allocating recycling credit within the foreground data, in order to replicate the method applied by Parker et al. (2007). Although this method is no longer considered appropriate for use in an attributional LCA, it was tested here to explain the discrepancy in results between the two studies.

The end-of-life recycling method (also known as the avoided burdens or closed-loop approximation method) is a method of allocating credit for the avoided production of primary material in the future by producing recyclable material (Schrijvers, et al., 2016). Recycled material consumed in the product life cycle, therefore, does not give an environmental credit so has the same burdens has primary material. The underlying mathematical expression for this method from Schrijvers et al. can be rearranged to form Equation 1, assuming that the impacts of the substituted primary material will be the same as the impacts of the consumed primary material and the quality correction factor is one (as for closed-loop recycling of a material such as steel):

$$E_{tot} = E_v + r(E_{RC} + E_{RRE} - E_v) + (1 - r)E_d \qquad (1)$$

where E_{tot} is embodied impacts per unit of material, E_v is embodied impact of primary material, E_{RC} is embodied impact of the recycling process, E_{RRE} is embodied impact of recovery and transport of the recyclable material, E_d is embodied impact of waste disposal and r is recycling rate at end-of-life. It can be seen that the first term E_v is the embodied impacts of all input material, which is considered to have the impacts of primary material. End-of-life impacts include the credit for recycling, described by $r(E_{RC} + E_{RRE} - E_v)$, which is a function of the difference between embodied impacts of the production of primary and recycled material. Disposal of non-recycled material is represented by $(1 - r)E_d$.

In order to simulate the method applied by Parker et al., the above method was applied only to the foreground data for steel. All background data was still sourced from ecoinvent v3.3, using the recycled content allocation method, as with the main analysis. Modifications were made as follows:

- A new input steel dataset was created by copying the ecoinvent v3.3 data for the global steel market, but replacing all flows of recycled steel with data for primary steel for the same region.

- Recycling credit was estimating by creating a waste flow with a global recycled steel market as input (as above, but with all primary steel replaced with recycled steel), and a global virgin steel market as avoided product.

**Impact Category**	**Recycled Content**	**APOS**	**Difference**	**Unit**
Climate change	35	35	0.9%	g CO_2 eq/kWh
Ozone depletion	3.7	3.9	-5.6%	µg CFC-11 eq/kWh
Photochemical oxidant formation	325	318	2.2%	mg PM10 eq/kWh
Terrestrial acidification	404	402	0.5%	mg NMVOC/kWh
Freshwater eutrophication	21	20	2.4%	mg SO_2 eq/kWh
Marine eutrophication	14	14	0.0%	mg P eq/kWh
Particulate matter formation	184	181	1.9%	g 1,4-DB eq/kWh
Human toxicity	33	33	0.2%	mg N eq/kWh
Terrestrial ecotoxicity	4.2	4.7	-9.6%	mg 1,4-DB eq/kWh
Freshwater ecotoxicity	906	947	-4.5%	mg 1,4-DB eq/kWh
Marine ecotoxicity	924	959	-3.7%	mg 1,4-DB eq/kWh
Ionising radiation	2.4	2.5	-4.8%	Bq ^{235}U eq/kWh
Agricultural land occupation	915	965	-5.5%	mm^{2}a/kWh
Urban land occupation	393	392	0.3%	mm^{2}a/kWh
Natural land transformation	8.5	8.1	4.2%	mm^2/kWh
Water depletion	241	248	-2.7%	cm^3/kWh
Metal depletion	26	26	1.9%	g Fe eq/kWh
Fossil depletion	10.0	9.7	3.2%	g oil eq/kWh
Energy	493	483	2.2%	kJ/kWh

Table S4.1 - Comparing results from the APOS and recycled content approaches to allocating for recycling

The result of running the analysis with this modification is a reduction in all impacts. Of the factors relevant for comparison with Parker et al.: climate change was found to be 28 g CO_2eq/kWh, cumulative energy demand 421 kJ eq/kWh and CO_2 emissions 26 g/kWh. This reduction is likely due to

the recycling rate of 90% being much higher than the average recycled content of the global steel mix in the ecoinvent data (43%) (ecoinvent, 2016).

Errors may have been introduced to this analysis by using a mixture of allocation methods, so use of the method described here is not recommended.

References

ABB (2001) Environmental Product Declaration - HD4. ABB T and D SpA - SACE T.M.S, http://search-ext.abb.com/library/Download.aspx?DocumentID=ITEPD008&LanguageCode=en&DocumentPartId=&Action=Launch,

ABB (2007) Environmental Product Declaration - Distribution transformer - 315kVA, 11kV, 3 phase, ONAN. BA Distribution Transformers, http://www.abb.co.uk/cawp/abbzh258/3d76091aeb235c70c12569ee002b47f4.aspx,

ABB (2010) Environmental Product Declaration - UniSwitch - Medium Voltage Equipment. ABB Power Distribution, http://www.abb.co.uk/cawp/abbzh258/3d76091aeb235c70c12569ee002b47f4.aspx,

Astudillo MF, Treyer K, Bauer C, Pineau P-O and Amor MB (2017) Life cycle inventories of electricity supply through the lens of data quality: exploring challenges and opportunities. The International Journal of Life Cycle Assessment 22:374-386. doi: 10.1007/s11367-016-1163-0

Axxiom (2008) Schmidt Bulk Abrasive Blaster Operation and Maintenance Manual, Axxiom Manufacturing Inc. Axxiom Manufacturing Inc, http://www.axxiommfg.com/dynamic/products/literature/20090806082022_Bulk_Abrasive_Blaster_2008.pdf. Accessed December 2008

Caterpillar (2011) Engine powered lift trucks Specifications 1.5 - 3.5 tonnes, Cat Lift Trucks, http://www.catlifttruck.com/ebrochures/english/Diesel/DP15-35N/CESC1356/index.html. Accessed December 2011

Classen M, Althaus H-J, Blaser S, Scharnhorst W, Tuschmid M, Jungbluth N and Emmenegger MF (2009) Life Cycle Inventories of Metals. Final report ecoinvent data v2.1. Swiss Centre for Life Cycle Inventories, Dubendorf, Switzerland,

ecoinvent (2016) ecoinvent database v3.3. Swiss Centre for Life Cycle Inventories, http://www.ecoinvent.org/home/,

Graco (2010) Graco Xtreme Packages - Instructions - Parts - 311164G, Graco, http://wwwd.graco.com/Distributors/DLibrary.nsf/Files/311164/$file/311164G.pdf. Accessed December 2011

Hempel (2007) Hempadur Glass Flake 35851/35853 Product Data Sheet, Hempel, http://www.hempel.dk/internet/hempelcomcopy3006.nsf/vALLBYDOCID2/15936EF96242F170C1256C3F00415AE8/$file/PDS_35851-35853.pdf. Accessed December 2011

Hempel (2010a) Safety Data Sheet - Hempadur Glassflake 35858. Hempel,

Hempel (2010b) Safety Data Sheet - Hempel's Curing Agent 97652. Hempel,

Jiven K, Sjobris A, Nilsson M, Ellis J, Tragardh P and Nordstrom M (2004) LCA-ship - Design tool for energy efficient ships - A Life Cycle Analysis Program for Ships. SSPA, TEM, MariTerm AB,

Kalpakjian S, Schmid SR and Kok CW (2008) Manufacturing processes for engineering materials. Pearson-Prentice Hall,

Parker RPM, Harrison GP and Chick JP (2007) Energy and carbon audit of an offshore wave energy converter. Proc IMechE Part A: J Power and Energy 221:1119-1130. doi: 10.1243/09576509JPE483

Schrijvers DL, Loubet P and Sonnemann G (2016) Developing a systematic framework for consistent allocation in LCA. The International Journal of Life Cycle Assessment 21:976-993. doi: 10.1007/s11367-016-1063-3

SWF (2011) SWF Krantechnik Technical Guide: Characteristics (SI/50Hz), Mannheim, Germany SWF Krantechnik GmbH, http://www.swfkrantechnik.com/html/bildarchiv/download/Produkt_Data/nova/SWF_NOVA_Tech-Guide_Characteristics_50Hz_09-2011.pdf. Accessed December 2011

Trelleborg (2009) Marine Products - Engineered Systems, Trelleborg Offshore, http://www.trelleborg.com/upload/TCL_TCI/docs/Trelleborg_Offshore_marine%20products.pdf. Accessed 25th January 2011

Weidema BP, Bauer C, Hischier R, Mutel C, Nemecek T, Reinhard J, Vadenbo CO and Wernet G (2013) Overview and Methodology - Data quality guideline for the ecoinvent database version 3 - ecoinvent report No. 1. Swiss Centre for Life Cycle Inventories, St Gallen,
